# Supplementary material for: Decreased tourism during the COVID-19 pandemic positively affects reef fish in a high use marine protected area
Source: PLoS One. 2023 Apr 12;18(4):e0283683. doi: 10.1371/journal.pone.0283683 (PMC10096236; doi:10.1371/journal.pone.0283683)
Supplement: S1 File — (DOCX) [file pone.0283683.s001.docx]

**Supporting Information: Decreased tourism during the COVID-19 pandemic positively affects reef fish in a high use marine protected area**

Kevin C. Weng^1*^, Alan M. Friedlander^2,3^, Laura Gajdzik^4^, Whitney Goodell^2,3^, Russell T. Sparks^4^

1 Virginia Institute of Marine Science, William & Mary, 1375 Greate Road, Gloucester Point, Virginia, USA

2 Fisheries Ecology Research Lab, Department of Biology, University of Hawai`i at Mānoa, Honolulu, Hawai`i, USA

3 Pristine Seas, National Geographic Society, Washington, District of Columbia, USA

4 Division of Aquatic Resources, Department of Land and Natural Resources, 1151 Punchbowl Street, Room 330, Honolulu, Hawai`i, USA

* Corresponding author, email: kcweng@wm.edu

**Supporting Methods**

**Human abundance**

**Table S1. Optimal model output summary for the variation in human abundance at Molokini through times.** Significant p-values are in bold.

|  | **Monthly number of visitors** | | |
| --- | --- | --- | --- |
| **Optimal model** | Visitors ~ s(month, bs=”cc’, k=7, by=pandemic) + s(time, bs=”cr’, k=12, by=pandemic) + pandemic | | |
| **Predictors** | **Estimates** | **p-values** | **Effective Degrees of Freedom (EDF)** |
| (Intercept) | 28360.8 | **2 x 10^-16^** |  |
| Smooth term (month x non-pandemic) |  | **2 x 10^-16^** | 4.062 |
| Smooth term, (month x pandemic) |  | 0.700 | 4.799x 10^-07^ |
| Smooth term (time x non-pandemic |  | 0.328 | 1.000 |
| Smooth term (time x pandemic) |  | **3.38 x 10^-6^** | 2.531 |
| Pandemic | 127224.8 |  | 0.614 |
|  |  |  |  |
| Observations | 108 | | |
| R^2^ | 0.824 | | |

**Fish habitat use and occupancy**

**Table S2. Fishes tagged with acoustic transmitters at Molokini**

| **Species** | **Specimen** | **FL (cm)** | **Date** |
| --- | --- | --- | --- |
| *Caranx melampygus* | CAME-1 | 44 | 26-May-20 |
| *Caranx melampygus* | CAME-2 | 38 | 27-May-20 |
| *Caranx melampygus* | CAME-3 | 49.5 | 27-May-20 |
| *Carcharhinus amblyrhynchos* | CAAM-1 | 93 | 27-May-20 |
| *Caranx melampygus* | CAME-4 | 37 | 28-May-20 |
| *Seriola dumerili* | SEDU-1 | 82 | 1-Jul-20 |
| *Carcharhinus plumbeus* | CAPL-1 | 112 | 1-Jul-20 |
| *Caranx ignobilis* | CAIG-1 | 94 | 1-Jul-20 |
| *Carcharhinus amblyrhynchos* | CAAM-2 | 101 | 1-Jul-20 |
| *Triaenodon obesus* | TROB-1 | 67 | 23-Jul-20 |
| *Seriola dumerili* | SEDU-2 | 70 | 5-Nov-20 |
| *Seriola dumerili* | SEUD-3 | 100 | 5-Nov-20 |

**Table S3. GLM model specifications and performance.** Formulations with lowest AIC are in bold.

| **Model structure (GLM, negative binomial)** | **Species** | **Zone** | **Diel period** | **AIC** |
| --- | --- | --- | --- | --- |
| daily_detections ~ daily_vessels | All | Both | All | 29192 |
| daily_detections ~ total_people | All | Both | All | 30217 |
| daily_detections ~ daily_vessels + ppb | All | Both | All | 29189 |
| daily_detections ~ daily_vessels * ppb | All | Both | All | 29178 |
| daily_detections ~ daily_vessels+ppb+species | All | Both | All | 27753 |
| **daily_detections ~ daily_vessels+ppb*species** | **All** | **Both** | **All** | **27667** |
| daily_detections ~ daily_vessels+ppb | CAME | Inside | Morning | 17204 |
| daily_detections ~ daily_vessels*ppb | CAME | Inside | Morning | 17206 |
| daily_detections ~ daily_vessels+ppb+individual fish | CAME | Inside | Morning | 16226 |
| daily_detections ~ daily_vessels*ppb+individual fish | CAME | Inside | Morning | 16228 |
| **daily_detections ~ daily_vessels+ppb*individual fish** | **CAME** | **Inside** | **Morning** | **16187** |
| daily_detections ~ ppb+daily_vessels*individual fish | CAME | Inside | Morning | 16189 |

# Interpretation guide for Figure 4


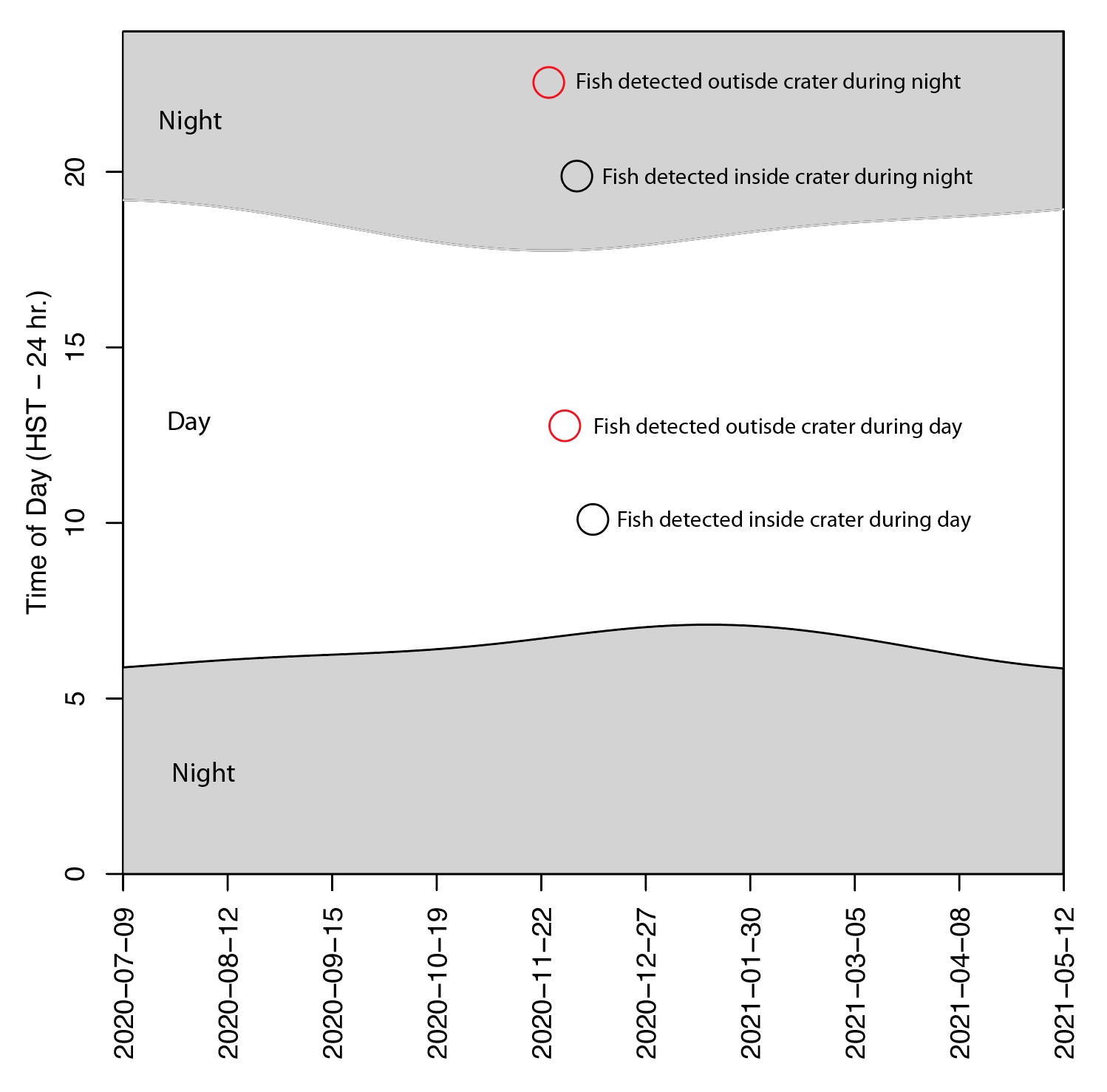


Figure S1. Interpretation guide for Figure 4, day-night plot of fish habitat use. Figure 4 of the main paper presents a timeseries of diel habitat use for a fish monitored using acoustic telemetry. The diel cycle is represented on the vertical axis, the white central region of the plot represents daytime, and the shaded upper and lower portions represent nighttime. The timeseries from May 2020 through May 2021 is represented by the horizontal axis. Circles denote detections of the fish, with red indicating detections outside of the crater in zone 2 where trolling is permitted, and black indicating detections inside the crater in zone 1 where all fishing is prohibited. Hence, a fish that remains inside the crater during day and goes to the outside at night would show black circles in the white shaded central portion, and red circles in the upper and lower grey shaded portions. A fish that was absent entirely from the MPA during morning hours would be indicated by a lack circles in a horizontal band within the white shaded region.
