# Supplementary figures and images for: Decreased tourism during the COVID-19 pandemic positively affects reef fish in a high use marine protected area
Source: PLoS One. 2023 Apr 12;18(4):e0283683. doi: 10.1371/journal.pone.0283683 (PMC10096236; doi:10.1371/journal.pone.0283683)

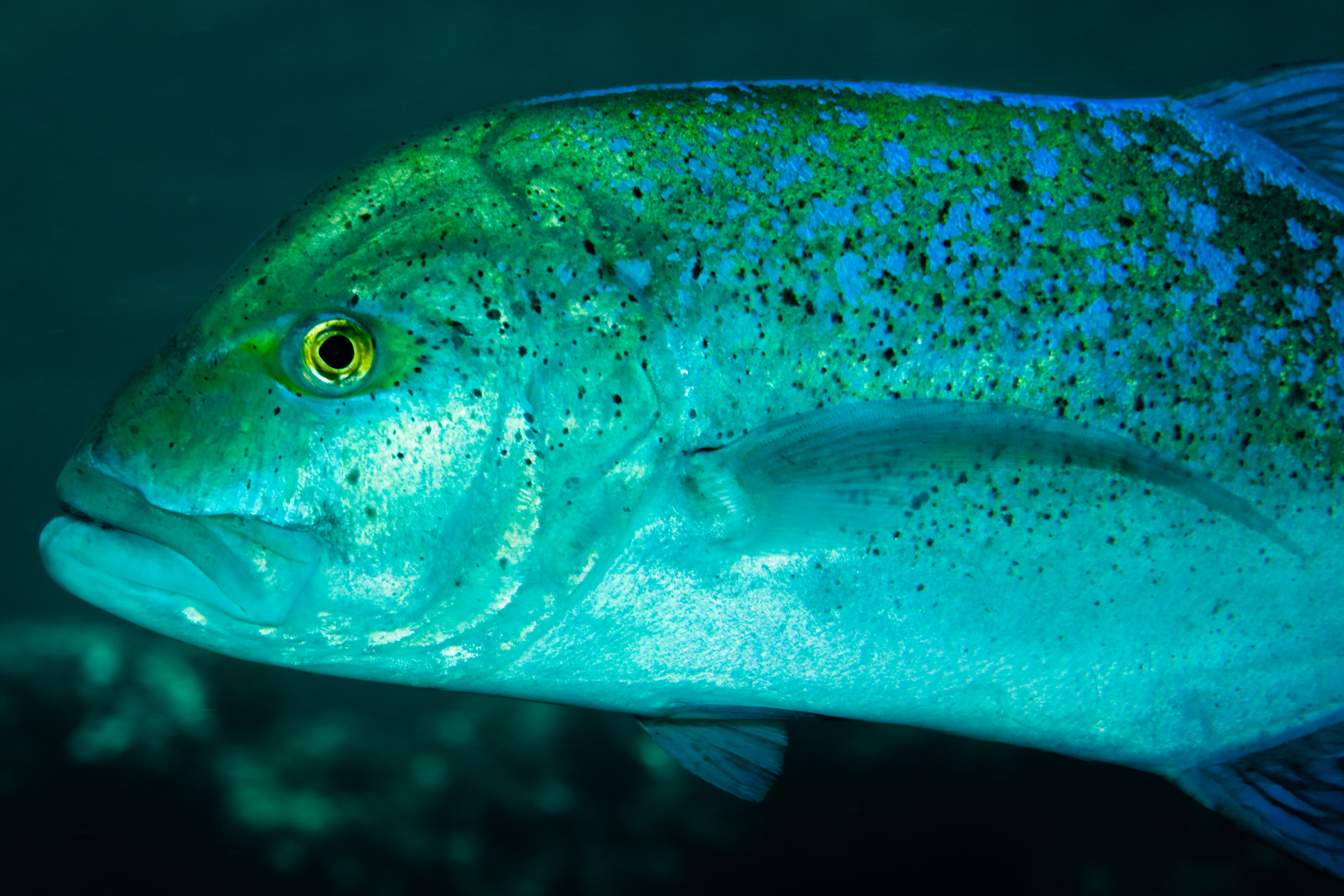

Supplement: S2 File — (JPG) [file pone.0283683.s002.jpg]
