# Supplementary material for: Decreased tourism during the COVID-19 pandemic positively affects reef fish in a high use marine protected area
Source: PLoS One. 2023 Apr 12;18(4):e0283683. doi: 10.1371/journal.pone.0283683 (PMC10096236; doi:10.1371/journal.pone.0283683)
Supplement: Attachment — Submitted filename: 20230202_responses to reviews.doc [file pone.0283683.s003.doc]

PLOS

1265 Battery Street, Suite 200

San Francisco, CA 94111

United States

2 Feb 2023

Reponses to Reviews for manuscript submitted as PONE-D-22-19263 "Decreased tourism during the COVID-19 pandemic positively affects reef fish in a high use marine protected area".

Kevin Weng, Virginia Institute of Marine Science, Virginia, USA (kcweng@wm.edu; 831-915-0296)

Alan Friedlander, Whitney Goodell, National Geographic Society, Washington DC, USA

Laura Gajdzik, Russell Sparks, Division of Aquatic Resources, Hawai`i, USA

Editor for original submission: Johann Mourier

| Comment (line numbers refer to original) | Response |
| --- | --- |
| EDITOR |  |
| If applicable, we recommend that you deposit your laboratory protocols in protocols.io | This project did not involve laboratory experiments. |
| 1. Please ensure that your manuscript meets PLOS ONE's style requirements, including those for file naming. | Amended |
| 2. We note that the grant information you provided in the ‘Funding Information’ and ‘Financial Disclosure’ sections do not match. | Amended |
| When you resubmit, please ensure that you provide the correct grant numbers for the awards you received for your study in the ‘Funding Information’ section. | Amended |
| 3. We note that you have stated that you will provide repository information for your data at acceptance. Should your manuscript be accepted for publication, we will hold it until you provide the relevant accession numbers or DOIs necessary to access your data. | Amended |
| 4. Please include your full ethics statement in the ‘Methods’ section of your manuscript file. In your statement, please include the full name of the IRB or ethics committee who approved or waived your study, as well as whether or not you obtained informed written or verbal consent. | At the end of section 2.4 we added "Fish tagging was conducted under an animal care protocol (IACUC-2020-10-05-14561-kcweng) approved by the William & Mary Animal Care and Use Committee (IACUC), USDA 52-R-0002, OLAW D16-00419 #A3713-01, and a Special Activity Permit from the State of Hawai`i (2021-28)." |
| 5. We note that Figure 1 in your submission contain [map/satellite] images which may be copyrighted. | The images and data used in Figure 1 are part of the public domain, and the caption has been revised to clarify this. |
| 6. Please review your reference list to ensure that it is complete and correct. If you have cited papers that have been retracted, please include the rationale for doing so in the manuscript text, or remove these references and replace them with relevant current references. Any changes to the reference list should be mentioned in the rebuttal letter that accompanies your revised manuscript. | Amended |
|  |  |
| REVIEWER 1 |  |
| Page 3, lines 40-45: Non-consumptive effects are defined in the abstract, but the definition is not clear in the intro. Also it is important to explain how these non-consumptive effects alter species composition and abundance. | The introduction to the paper has been revised to include more references to the literature, including changes in community structure and abundance. |
| Page 3, line 58: Not sure what you mean by “more pristine biomass” | Given the negative impacts resulting from tourism (Grorud-Colvert et al., 2021), large reductions in human abundance are expected to allow wildlife to reestablish habitat use (Frid and Dill, 2002), resulting in higher biomass particularly at higher trophic levels. |
| Page 3, lines 60-61: Are these numbers referring only to tourist related activities? | These numbers are for the travel and tourism sector. We had a mistake in allocating it to the US. The numbers actually refer to all countries. The line now reads " The COVID-19 pandemic caused a substantial decline in tourism (Gössling et al., 2021). Global economic losses in the travel and tourism sector are in the range of USD 4 to 12 trillion in gross domestic product, 164 to 514 million jobs, and USD 363 to 1134 billion in capital investment (Škare et al., 2021)." |
| Page 4, lines 74-75: This sentence is not clear. Maybe you can talk about number of visitors instead of margin styles... | We have eliminated the jargon, so the line now reads "At a given level of economic benefit, ecological impacts are likely to be lower when tourism focuses on premium experiences with fewer visitors, each spending more money, instead of minimizing costs to maximize visitor numbers (Gössling et al., 2016). " |
| Page 5, line 94: “allowed trolling only on the outside.” | On the outside of the crater, all fishing methods are prohibited exept trolling. The line now reads "The Molokini Shoal MLCD created a two-zone management system that prohibited all fishing inside the crater and allowed only one method, trolling, on the outside of the crater." |
| Page 6, lines 115-116: There is very little mention of habitat use in the intro. What is the relation between habitat and fish biomass? How do fish use certain habitats? Are there studies contrasting habitat use patterns of fish across gradients of human pressure? Do we have information of fish habitat use in pristine (semi-pristine) environments? We (the reader) need more information and background to justify the hypothesis and perhaps some predictions on what you would expect to find. | In response to these framing questions, we have broadened our references to existing literature, thereby providing greater context with regards to habitat use and the impacts of human presence on fish habitat use. Please see the first three paragraphs of the introduction, which have been revised and expanded, and now cite key papers on fish responses to human presence. |
| Page 5, line 119: “Insights gained from this study may inform future management strategies” | We have revised the line to read "Insights gained from this study will inform 30x30 planning and are relevant to three of the four priorities in the Molokini conservation action plan" |
| Page 7, lines 144-145: Not sure that I understand the difference | The paragraph has been revised for clarity. |
| Page 8, lines 155-156: “identified all fishes visible within 2.5 m to either side of the centerline (125 m2 transect area)” | The paragraph now reads "A scuba diver swam a 25 x 5 m transect at a constant speed, identified all fishes visible within 2.5 m to either side of the centerline (125 m2 transect area) to the lowest possible taxon, and estimated the total length (TL) of fishes to the nearest centimeter." |
| Page 8, line 158: Remove “Yet” | Removed |
| Page 8: lines 154-162: It would be interesting to have some knowledge of where these transects were conducted so that we have some sense of what was the total area that was covered. | We have included a figure in the supplement to show the locations. |
| Page 9, line 174: “if a fish was inside or outside of the crater” | Revised accordingly |
| Page 9, line 186: what species and how many individuals from each species were tagged? Why these species were important to answer your question/hypothesis? I think this is something that the intro/methods should explore more. Are you tagging only predatory fishes? Why? In the results there is only information of C. melampygus. | We have included a table of tagged fishes in the supplement. Furthermore, we have explained the importance of direct and indirect effects, and that predators are important in mediating indirect effects. We have added text to both the introduction and the methods. |
| Page 10, lines 196-197: The model needs more explanation. What is Y in the equation (I am assuming is human abundance, but it can be clearer in the text)? Why using cos? how did you compare and selected models? How you assess the fit and model performance? What software and/or R functions did you use for this procedure? | The model used in the first submission was a sine wave to capture the seasonality in the number of visitors in Molokini. We had increased the complexity of the models by adding a sin and cos that varied pre-pandemic and during pandemic. However, these models did not account for possible autocorrelation in our time-series dataset. Therefore, in this revised version of the MS, we explored generalized additive models (GAM) and generalized additive mixed models (GAMM) with an autoregressive-moving-average (ARMA) residual autocorrelation structure. The models were generated in R using the package mgcv. We selected the best model by comparing the AIC values and chosed the simplest model when the lowest AIC values did not differ >2. Additional details are provided in the methods. Most importantly, both our first model and the GAM provide similar results: seasonal increase in the number of visitors during summer times every year except during the COVID-19 pandemic when the Molokini MLCD received little to no visitation. |
| Page 10, line 204: You only included year as a factor in the model or you also account for other factors (depth, temperature, season, etc)? It would be important to include other factors available in the model that could influence fish distribution and abundance. Also, did you use data from both fish survey protocols for this? Yes/No, why? | There is not enough resoltion to anayze these using the proposed factors.  The two fish survey datasets were analyzed separately. The text has been revised to clarify. |
| Page 10, line 205: why Poisson distribution? Can you expand on this? | Poisson is the standard approach for count data. |
| Page 11, line 214: What is a sampling period? Also, it would be good to have an idea of what a full/complete model looks like and to all the candidate models that you consider, as well as the model that was selected and the evidence for that specific model. | Sampling periods are explained in the revised methods. We used AIC to determine distribution type. |
| Page 11, line 216: Why? It seems to me that transect is the sampling unit that you are using so not sure why it is being considered a random effect in the models? | The model has been revised so that transect is not considered a random effect. |
| Page 11, lines 226-228: This needs to be further explained. How exactly did you combine these data? | Our wording in the original version was confusing. We did not actually combine the fish data and the vessel data. Instead, we used fish data as the response variable and vessel data as the explanatory variable. The text now reads "Fish tracking data were downloaded from the passive acoustic monitoring array, processed in the manufacturer supplied software (Vue, Vemco Ltd.) and imported to R. The fish movement data were used as the response variable. Vessel and human abundance data from the DAR database were used as the explanatory variable." |
| Page 11, lines 233-234: What not using a predictor of human activity that could combine all these 3 variables instead? | There is a problem of colinearity between 'daily count of people' and 'daily count of vessels' so including both of these in a single model is contraindicated. In the revised version of the paper we have added the evaluation of colinearity using 'variance inflation factors (VIF)' to the methods to explain our model selection. |
| Page 13, lines 266-267: giant travally and whitetip reef shark are not top predators, they are mesopredators. Giant travally is a large mesopredator and whitetip reef shark is a small mesopredator. It would be important to adecuately define what a top predator is as there is a lot of evidence that suggest that both species are actually mesopredators rather than top predators. | We have removed the use of "top predator" from the manuscript, and instead simply use the term "predator". We feel that a detailed treatment of different levels of predators would not be relevant to the paper. |
| Page 14, line 278: Is this refers to the biomass of all species or predator species or top predators (based on your text)? This is not clear. | We have amended the first line to read "Comparisons of fish biomass at all trophic levels among sampling periods ..." |
| Page 14, line 290: More information about the models is needed. i suggest having a table with the full model and candidate models that were evaluated as well as their support | The supplementary material includes the model results for the full dataset as well as the subset for inside during morning (for the best performing model). We have also added a table that shows all candidate models. |
| Page 15, line 299: This panel is confusing. There is a lot going on and the patterns are not clear. | We have amended the text of the figure legend for clarity, and have produced a supplementary figure that provides guidance for interpretation of the figure. |
| Page 16, line 322: “resulting cessation of tourism resulted in a significant rebound of biomass during April 2020” | It is not clear what changes the reviewer is requesting. |
| Page 18, line 370: Maybe you can move this text at the beginning: “Understanding the impacts of non-consumptive uses such as tourism is critical to statewide marine spatial planning, improving effectiveness of existing MPAs, and implementing a statewide network of MPAs.” | It is not clear why the text should be moved. |
|  |  |
| REVIEWER 2 |  |
| Abstract |  |
| - Recommend that the authors substitute the word ‘consumptive’ with ‘ extractive’ throughout the manuscript. Consumptive implies that a resource is eaten or consumed. Fishing activity isn’t always consumptive, but does result in extraction from a community. My experience is that extractive is a more commonly used term in the literature. | It depends on the context of the study. When the question concerns animal behavior across a gradient of predation risk, 'consumptive' and 'non-consumptive' are the most commonly used terms in the literature. When the question concerns alternate economic approaches to the monetization of natural resources, 'extractive' and non-extractive' are used. While both of these contexts are relevant to our study, the key question is the response of animals to different risk levels. Hence, we use the term 'non-consumptive'. |
| - Ln 30: reword sentence to read ‘change community composition and habitat usage at local scales...’ | Sentence has been modified, please see response to next comment. |
| - Ln 31: I don’t think your findings are strong enough to say that you have evidence that tourism ‘can compromise ‘ ecosystem function. Please reword to ‘could impact’ ecosystem function. | Agreed, the word "could" has been included. The sentence has been reworded as "These displacement effects change community composition and biomass, which could affect key processes such as spawning, foraging and resting, and have knock-on effects that compromise ecosystem function and resilience." |
| Introduction |  |
| - As above, recommend changing ‘consumptive’ to ‘extractive’ throughout. | See prior comment. |
| - Ln 42: Recommend changing ‘humans’ to ‘human activity.’ Fish won’t necessarily modify behaviour to humans themselves, but rather the activities that they conduct. As an example, vessel movement may alter behaviour. | Revised accordingly |
| - Ln 57: Change to ‘negative impacts that can occur as a result of tourism.’ Not all tourism is detrimental or modifies behaviour in a significant way. | Revised accordingly |
| - Ln 57: change ‘abundance’ to ‘activity.’ | Revised accordingly |
| - Ln 58: change to ‘re-establish optimal habitat use.’ The fact that the fish are still around indicates that they are still utilising the habitat, just not in their preferred way. | Revised accordingly |
| - Ln 67-84: Some of the content of these two paragraphs is likely better suited to your discussion. At the moment your intro is quite lengthy. | We feel it is important that readers are aware of the 30x30 Initiative to frame their understanding of the work in the context of the UNEP CBD. We have revised the first sentence to read "In response to ongoing environmental degradation, the State of Hawai`i is developing a Marine 30x30 Initiative, in the context of the United Nations Convention on Biological Diversity, which aims to establish management over 30% of Hawai`i’s nearshore waters by 2030 (https://dlnr.hawaii.gov/dar/30x30/)."  Lines 78-84 have been moved to the discussion. |
| - Ln 111: Change to ‘displaced from inside the’ | Revised accordingly |
| Methods |  |
| - Ln 154: What is the experience level of divers/observers conducting surveys and how is quality control ensured? Were divers/observers consistent between temporal surveys? Is there the potential that there was any observer bias associated with the 2004-2020/21 survey differences? | We have added the line "To ensure consistency within surveys, FHUS dives were conducted by AMF and WG, while FAHU surveys were conducted by the DAR survey team directed by RTS." |
| - Ln 155: Does this include cryptic species? | No, cryptic species are not included. As the methods state, only fishes visible to the diver are recorded. |
| - Ln 169: How long was the telemetry data recorded for (i.e. start and finish dates)? | We have added the text "covering May 2020 through May 2021." |
| - Ln 186: Please provide more detail on species tagged in this experiment. Its as much of an interest to know which species didn’t modify behaviour, as those that did. | We have included a table of tagged fishes in the supplement. |
| - Ln 204: Is there supposed to be a sub heading for this paragraph, as it doesn't appear to fit under the previous heading | We have added the subheading "2.5.2 In situ fish surveys" |
| - Ln 207: Did you consider running a PERMANOVA to statistically examine shifts in community structure? Given that you state that there was a shift in community composition as one of your conclusions, I feel a statistical test is warranted. When combined with a CAP analysis, would enable you to identify what species are statistically responsible for the shifts. | We have included the PERMANOVA as requested. Comparisons of fish assemblage structure among sampling periods based on biomass were investigated for both FHUS and FAHU surveys using permutation-based multivariate analysis of variance (PERMANOVA). Bray–Curtis similarity matrices were created from biomass of fish taxa. Prior to analyses, fish biomass was square root transformed. Interpretation of PERMANOVA results was aided using individual analysis of similarities (ANOSIM). PCO is along the same vein as CAP so it would be redundant to do both. |
| - Ln 213: As above with regards to the PERMANOVA and CAP analysis. | See above comment. |
| Discussion |  |
| - While interesting, the discussion is highly focused on management recommendations. While this should definitely be included in your discussion, I feel that it could be refocused a little to also include discussion of the potential ecosystem effects of your findings. In particular, I would like to see some discussion of the potential trophic and ecosystem function effects of your findings. Your primary effects have been on higher order mobile predator species, ironically the same groups that tend to be highly targeted by fishing activity. As such, high intensity tourism may well be having similar effects at local scales as low intensity fishing (i.e. fishing effort at a level that primarily only effects higher order groups). This is actually a really interesting finding and worth discussing in more detail. | Thank you for this excellent suggestion. We have added a paragraph to the discussion pertaining to the ecosystem effects of predator displacement. |
| - Ln 332: Change to ‘on this group of fishes.’ | Modified accordingly |
| - Ln 387: I disagree with this statement. You haven't examined habitat at all. What you have shown is that high tourism can affect the space usage of some species, particularly higher order predators. This could have repercussions for trophic links and ecosystem function akin to the effects of fishing (which also tends to target higher order group). | The sentence has been modified to read "the business-as-usual conditions of high tourism alter community structure by displacing predatory fishes to deeper environments" |
|  |  |
| REVIEWER 3 |  |
| Line 62: This is an interesting example, but perhaps the authors could be more specific about the drivers of this increase in fish abundance (i.e. Lecchini et al. (2021) found beavhioural changes that lead to increased measures of fish biomass instead of actual changes in population density). A few linking words could also help the flow of this paragraph. Maybe something like “In French Polynesia, this decrease in tourism resulted in an associated change in fish community structure, where surveys revealed a short term increase in fish biomass in the absence of tourism”. | Lecchini et al reported large increases in the biomass of fishes during the period when tourists were absent, with the increases being larger in tourist sites than in control sites. After the lockdown was lifted, fish biomass returned to pre-pandemic levels. The values reported by Lecchini are "number of fish / m2", meaning that any changes reported are real changes in density. Lecchini attributed these changes to displacement of some individuals away from sites visited by humans under business-as-usual conditions, and then a immigration of fishes to these sites during the lockdown. As such, the reviewer's statement "beavhioural changes that lead to increased measures of fish biomass instead of actual changes in population density" is incorrect. The reviewer is implying that the behavioral changes altered the catchability of fishes by diver visual census, which is not an explanation raised by Lecchini et al. We do feel that the reviewer's suggested wording is helpful so we have revised the sentence to read "In French Polynesia, the absence of tourism during a 45 day lockdown resulted in a short term increase in fish biomass". |
| Line 65: I would leave this last line a bit more open to highlight a knowledge gap rather than make predictions/hypothesis in the middle of the introduction. | We have revised the sentence to read "so a resurgence of wildlife at tourist sites was possible." |
| Methods: generally these seem appropriate for the questions being asked. The sampling gap between 2004 - 2021 is not ideal, but I understand that sampling is rarely perfect when using historical data sets. A table of the tagged species would be useful. This would help the reader understand sample size, what species have been tagged, and is meant exactly by the term “predatory fishes”. Depending on how many different species were tagged, the differences in their ecology (home ranges etc) and the degree to which they are targeted by fishers, it could be interesting to separate these species in the analysis. | We have included a table of tagged fishes in the Supplement. Due to the low sample size for some species we did not conduct statistical analysis. |
| Results: It is honesetly very hard to comment on the results because I cannot read the graphs. Discussion: |  |
| I think the Discussion makes som excellent and interesting points. I do wonder whether the impact of displacing predators from shallow reefs cascades down to lower trophic levels. There are a few examples from the terrestrial literature of humans changing predator behaviour, which subsequently benefits lower trophic levels (e.g. Suraci, Justin P., et al. "Fear of humans as apex predators has landscape‐scale impacts from mountain lions to mice." Ecology Letters 22.10 (2019): 1578-1586.). Could be something to consider adding into the discussion. | We have incorprated this reference into the discussion in the paragraph pertaining to the ecological effects of predator displacement. |
| Line 305-313: the first paragraph of the Discussion seems more suited to the Introduction as there are no results or discussion of these findings. I would suggest starting with paragraph 2 (which is a really interesting). | We have removed paragraph 1 of the discussion as recommended. |
| Line 332: Line 332- The authors make a good point about noise disturbing the fish (although some references would be good here). In addition to noise, the presence of humans could also displace fish. If fish encounter spearfishers during their movements, they can learn that humans represent a potentially lethal threat, especially species that are targeted by these fishes, and flee from these fishers | We have included a reference |
| These reference could be useful: | We have elected not to include additional disucssion and references for the sake of brevity. |
| Goetze, Jordan S., et al. "Fish wariness is a more sensitive indicator to changes in fishing pressure than abundance, length or biomass." Ecological Applications 27.4 (2017): 1178-1189.). |  |
| Januchowski-Hartley, Fraser A., et al. "Fear of fishers: human predation explains behavioral changes in coral reef fishes." PLoS One 6.8 (2011): e22761. |  |
| Januchowski-Hartley, Fraser A., Kirsty L. Nash, and Rebecca J. Lawton. "Influence of spear guns, dive gear and observers on estimating fish flight initiation distance on coral reefs." Marine Ecology Progress Series 469 (2012): 113- 119. |  |
| Line 352: should this be “our findings suggest” ? | We have rewritten this paragraph for clarity and brevity. |
| Line 354: what aspect of visitor education could be enhanced? | We have removed the reference to visitor education. |
| Line 368: be specific about what is meant by the “health” of the MPA | We have changed the sentence to read "greatly reducing human impacts on the MPA" |

References Cited

Frid, A., and Dill, L., 2002, Human-caused disturbance stimuli as a form of predation risk: Ecology and Society, v. 6, no. 1.

Gössling, S., Ring, A., Dwyer, L., Andersson, A.-C., and Hall, C. M., 2016, Optimizing or maximizing growth? A challenge for sustainable tourism: Journal of Sustainable Tourism, v. 24, no. 4, p. 527-548.

Gössling, S., Scott, D., and Hall, C. M., 2021, Pandemics, tourism and global change: a rapid assessment of COVID-19: Journal of Sustainable Tourism, v. 29, no. 1, p. 1-20.

Grorud-Colvert, K., Sullivan-Stack, J., Roberts, C., Constant, V., Costa, B. H. e., Pike, E. P., Kingston, N., Laffoley, D., Sala, E., Claudet, J., Friedlander, A. M., Gill, D. A., Lester, S. E., Day, J. C., Gonçalves, E. J., Ahmadia, G. N., Rand, M., Villagomez, A., Ban, N. C., Gurney, G. G., Spalding, A. K., Bennett, N. J., Briggs, J., Morgan, L. E., Moffitt, R., Deguignet, M., Pikitch, E. K., Darling, E. S., Jessen, S., Hameed, S. O., Carlo, G. D., Guidetti, P., Harris, J. M., Torre, J., Kizilkaya, Z., Agardy, T., Cury, P., Shah, N. J., Sack, K., Cao, L., Fernandez, M., and Lubchenco, J., 2021, The MPA Guide: A framework to achieve global goals for the ocean: Science, v. 373, no. 6560, p. eabf0861.

Škare, M., Soriano, D. R., and Porada-Rochoń, M., 2021, Impact of COVID-19 on the travel and tourism industry: Technological Forecasting and Social Change, v. 163, p. 120469.
